# Influence of Controlled Cooling on Crystallinity of Poly(L-Lactic Acid) Scaffolds after Hydrolytic Degradation

**DOI:** 10.3390/ma13132943

**Published:** 2020-06-30

**Authors:** Javier Vazquez-Armendariz, Raquel Tejeda-Alejandre, Aida Rodriguez-Garcia, Yadira I. Vega-Cantu, Christian Mendoza-Buenrostro, Ciro A. Rodriguez

**Affiliations:** 1 Tecnológico de Monterrey, Escuela de Ingeniería y Ciencias, Monterrey 64849, Mexico; A00824090@itesm.mx (J.V.-A.); raquel.tejeda@tec.mx (R.T.-A.); yadira.vega@tec.mx (Y.I.V.-C.); 2 Laboratorio Nacional de Manufactura Aditiva y Digital (MADIT), Apodaca 66629, Mexico; 3 Universidad Autónoma de Nuevo León, Facultad de Ciencias Biológicas, Instituto de Biotecnología, Ave. Pedro de Alba S/N, Ciudad Universitaria, San Nicolás de los Garza 66455, Mexico; aida.rodriguezgrc@uanl.edu.mx

**Keywords:** polymer crystallinity, bimodal scaffolds, fused deposition modeling, hybrid manufacturing, additive manufacturing

## Abstract

The use of hybrid manufacturing to produce bimodal scaffolds has represented a great advancement in tissue engineering. These scaffolds provide a favorable environment in which cells can adhere and produce new tissue. However, there are several areas of opportunity to manufacture structures that provide enough strength and rigidity, while also improving chemical integrity. As an advancement in the manufacturing process of scaffolds, a cooling system was introduced in a fused deposition modeling (FDM) machine to vary the temperature on the printing bed. Two groups of polylactic acid (PLA) scaffolds were then printed at two different bed temperatures. The rate of degradation was evaluated during eight weeks in Hank’s Balanced Salt Solution (HBSS) in a controlled environment (37 °C–120 rpm) to assess crystallinity. Results showed the influence of the cooling system on the degradation rate of printed scaffolds after the immersion period. This phenomenon was attributable to the mechanism associated with alkaline hydrolysis, where a higher degree of crystallinity obtained in one group induced greater rates of mass loss. The overall crystallinity was observed, through differential scanning calorimetry (DSC), thermo gravimetric analysis (TGA), and Fourier transformed infrared spectroscopy (FTIR) analysis, to increase with time because of the erosion of some amorphous parts after immersion.

## 1. Introduction

### 1.1. Justification

The role of bone in the musculoskeletal system is significant as an element providing a supporting structure, for storing nutrients, and for safeguarding vital organs, among other functions. While bone possesses a remarkable regenerative ability, it can fail on healing under variable and insufficient conditions [1]. These conditions can result from several causes, including fractures and bone defects. When the patient’s system is not able to generate enough bone to repair these conditions, invasive techniques such as a bone graft are needed to act as a space filler or as a scaffold for new bone generation. Frequently, tissue from a patient is harvested for bone grafting, this is called an autograft. When the tissue is harvested from donors or cadavers, and is then used, it is called an allograft. In the field of reconstructive orthopedics, bone repairs are made to correct skeletal defects and accelerate bone healing. In addition, bone grafts often bring enhanced mechanical and biological function [2].

The bone graft market is segmented into allografts and synthetic materials. The allografts consist of demineralized bone matrix, while the synthetic grafts include scaffolds derived from ceramic, composite, polymeric, or other materials. The global bone graft and substitutes market was worth US $2.6 billion in 2018, with the allograft market holding the largest revenue share [3].

The clinical problems related to grafts for bone defects [4], in addition to the limited current solutions, have encouraged research to seek new approaches for bone grafting. One important task is to design scalable and complex geometric structures that possess the optimal physical properties to allow for the transmission of regeneration and vascularization forces. This task must be accomplished while at the same time providing enough strength and rigidity during the regenerative process [4]. Bone repair and regeneration can be improved using biocompatible and biodegradable scaffolds possessing comparable mechanical properties to bone and controlling the degradation properties. Recent advances in the design and manufacturing of scaffolds, consider the use of hybrid manufacturing. It involves the use of additive manufacturing to create balanced structures in pore size: macro pores arranged in lattice shapes that contain meshes with nano pores formed, for example, by electrospinning. The combination of both pore sizes provides an optimal micro and nano structure for cell adhesion and growth by increasing the area available for cell penetration. In hybrid manufacturing, some of the most important variables to be modulated are temperature of plate, heating temperature, and printing velocity. Depending on those manufacturing variables, different mechanical and chemical properties in scaffolds can be obtained, as well as changes on degradation rate of the material. This is another aspect that is necessary to be considered, since the scaffold gradually must be replaced by the regenerated tissue.

The materials and scaffolds used in bone tissue engineering should meet several specific characteristics: (i) biocompatibility, or the ability to accomplish the function without an immune reaction from the host; (ii) biodegradability, that is the capacity to decompose when, and as, new bone is produced, and (iii) structural features, including porosity and suitable osteoinductive/osteoconductive properties, to encourage cellular proliferation and osteogenic differentiation at the healing site [5]. The synthetic bone graft must simplify the regeneration and remodeling of the bone tissue, while progressively resorb in reaction to the new bone structure.

Additional considerations are required, including the evaluation of the release kinetics of the drugs used for regeneration of the damaged tissue, and the analysis of the degradation products. These products are derived from an in vivo interaction with some of the materials of the scaffold (such as PLA). Each of these aspects is important to be understood because body fluids are known to reduce local pH and induce inflammatory reactions [6] when slow-degrading polymers (degradation/resorption >2 years) are involved in long-term in vitro or in vivo studies [7].

### 1.2. Related Work

Porous bone scaffolds can be produced by different methods, including electrospinning [8], phase separation [9], and freeze-drying [10]. However, the structural features such as pore size, geometry, and interconnectivity cannot be fully controlled by these approaches. Additionally, scaffolds which reflect personalized porosity and geometry for site-specific defects are difficult to construct [11]. Additive manufacturing (AM) offers the possibility for these scaffolds to be designed and fabricated using innovative technologies [1]. Different AM approaches, or the combination of them (hybrid manufacturing), allow for the construction of complex geometries for scaffolds directly from a computer aided design (CAD) file. In hybrid manufacturing, some of the most important variables to be modulated are the temperature of the bed, the heating temperature, and the printing speed. Depending on those manufacturing variables, different mechanical and chemical properties for the printed scaffolds can be obtained, as well as changes in the degradation rate of the product. This is another aspect that must be taken into account, since the scaffold gradually must be replaced by the regenerated tissue during the healing process [12].

PLA is the one of the commonly used biodegradable polymer in biomedical applications due to its favorable biocompatibility and to its safe degradation products [13,14,15,16]. Clinical applications of PLA include: (a) inguinal hernia repair meshes, such as ProGrip™ from Medtronic [17], which is the most used self-gripping mesh made of hydrophilic monofilament polyester (PET) knit with resorbable polylactic acid (PLA) microgrips [18], (b) sutures [19], (c) drug delivery systems [20], (d) orthopedic surgery screws [21], and (e) bone implants [22]. The PLA biomedical scaffold implants have been comprehensively studied with in vitro and in vivo tests to analyze the degradation time and proper conditions to reach it, using different animal models, such as rabbits [23] or rats [24], as well as different types of bone defects. Properties like high tensile strength, elongation, and modulus make this material suitable for orthopedic fixation [25].

The behavior of PLA is restricted to aging mechanisms, such as thermal decomposition, hydrolysis, oxidation, and natural weathering. The degradation rates depend on the crystallinity of PLA, the molecular weight distribution, morphology, and the water diffusion rates into the polymer. Harris et al. [26] discussed the lack of studies on the chemical degradation of PLA, and it was shown that under atmospheric conditions, the degradation rate of PLA was slow [27]. Table 1 summarizes the most relevant studies related to changes in characteristics and crystallinity of some 3D printed scaffolds. There are several studies that use PLA for both, degradation and crystallinity analysis.

Next, we describe relevant studies focused on improved mechanical properties of base materials and scaffolds. Yang et al., for example, designed a temperature control into the fused deposition modeling (FDM) process of poly-ether-ether-ketone (PEEK) to achieve different degrees of crystallinity [33]. PLA has been subjected during the printing process to a continuous heat transfer, which showed a significant increase in the degree of crystallization for in-process treated samples [34]. In a related study concerning the printing process of PLA, a reduction in degradation temperature and molecular weight was achieved since the printing process induced a shortening in polymer chains. However, crystallinity percentage was similar before and after printing, 23% and 24%, respectively [14]. Furthermore, Benwood et al. analyzed the effect on the mechanical properties of PLA after varying the thermal conditions during the printing process. Their results showed an increase in mechanical properties related to the content of the PLA crystalline phase [36].

The influence of implementing a controlled cooling system in the manufacture of PLA bimodal scaffolds using the FDM approach was previously demonstrated [11,37]. Lara-Padilla et al. recently showed a clear influence of the cooling condition in the mechanical properties of scaffolds [11]. When using a cooling system, both scaffold stiffness and yield strength presented a decrease of 38% and 30%, respectively. It would be an interesting progress to know if a cooling system can be introduced to control the temperature of the printing bed. Even more importantly, if the scaffolds printed with this system would have a change in degradation rate through the mere modification of such temperature, recognizing that the appropriate characterization of temperature profiles, degradation tests, and analyses are still necessary (see Table 1).

### 1.3. Objective

This work describes the introduction of a new cooling system to be used during the printing process of PLA scaffolds. In this study, the objective was to explore the effect of temperature variation during the printing process on the degradation rate of the scaffold as a consequence of alterations in the degree of polymer crystallinity. To evaluate this hypothesis, two groups of PLA scaffolds were produced through FDM at two different printing bed temperatures. Then, both groups were immersed during eight weeks in HBSS solution in a controlled environment. Crystallinity assays were carried out to evaluate the degradation of the polymer.

## 2. Materials and Methods

### 2.1. Polymer

The scaffolds were printed using a commercial 3D green filament based on polylactic acid (PLA, eSUN^®^, Hong Kong SAR, China) of 1.75 mm diameter with a melting temperature of 180 °C (Table 2). One of the main advantages of using this polymer is its biocompatibility properties. This was confirmed in previously reported studies where the same commercial PLA filament did not show a cytotoxic effect on primary human bone marrow cells and MC3T3-E1 cells [14,15,16]. According to the safety data sheet of PLA manufacturer, the filament is composed by a 98% of polylactide resin and a 2% of calcium carbonate [38]. No information about the nature of the green pigment or other additives is provided.

### 2.2. Manufacturing Process

The bimodal scaffolds used to conduct this study were manufactured in a hybrid printer. The printer can use different types of polymers, among which PLA and polycaprolactone (PCL) stand out, and it has a resolution of approximately 0.5 mm. In addition, it has two proportional-integral-derivative control systems (PID), one for the extrusion temperature and another to obtain a cooling control in the printing base and in the electrospinning process. The control of the stepper motors used for the movement in the X, Y, and Z axes was provided by Aerotech^®^ MP drivers (Pittsburgh, PA, USA).

The printer control software was designed in LabVIEW v2013 (National Instruments, Austin, TX, USA). The use of this software allows the integration of various technology platforms, such as the Aerotech^®^ MP drivers and Arduino^®^ (Piedmont, Italy). The design of the scaffolds was performed using SolidWorks 2019 (Dassault Systèmes, Waltham, MA, USA) with the purpose of obtaining the points and cartesian coordinates required for the structuring of the G code. This code was introduced in the control program of the printer for subsequent manufacture.

**Figure 1:**
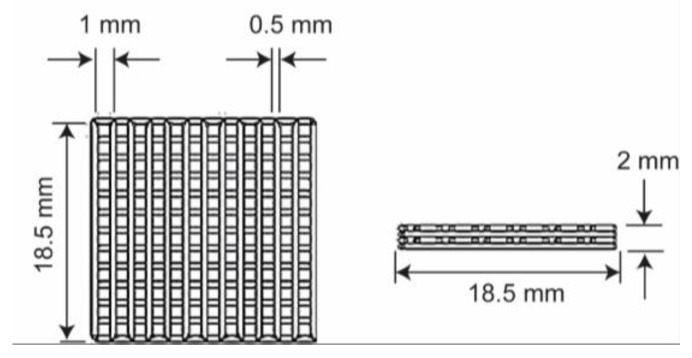
Polylactide acid (PLA) experimental sample dimensions in front and lateral views. Source: author.

The scaffolds used for the study were fabricated in a four-layered square form of 18.5 mm on the side with 1 mm as pore size, and 0.4–0.5 mm as the layer height (30% of porosity), as shown in Figure 1. A total of 48 PLA scaffolds were printed using a cooling stage (SCF-C group) and a non-cooled stage (SCF-NC group) for further crystallinity studies. The room temperature and the relative humidity during the printing process were 25 °C and ~45%, respectively; the extruder temperature was settled at 200 °C and the printing velocity at 8 mm/s. After the printing, the specimens were ultrasonically cleaned with isopropanol of analytical grade (CTR, Monterrey, Mexico) and then dried at room temperature for two hours. Scaffolds were grouped into eight triplicates for the SCF-NC and SCF-C groups, then individually labeled and weighed using an analytical balance (Sartorius CPA323S, Göttingen, Germany).

### 2.3. Thermal Analysis of the Printing Process

The temperature of the bed was measured in order to know the capability of the cooling system. Analysis of the temperature during the printing was provided in real-time by a thermal infrared camera (FLIR C3, Wilsonville, OR, USA). It was placed above the printing bed at 25 cm from the scaffold. In this way, the distribution of cooling on the upper surface of the scaffold was analyzed. Thermal imaging allowed an evaluation of the degree of damage produced during the printing in the subsequent layers. It also served as a method for comparing the temperature profile when printing, with and without the cooling stage.

### 2.4. Immersion Test

The degradable behavior of the PLA scaffolds was evaluated during eight weeks in Hank’s balanced salt solution (HBSS) (CTR Monterrey, Mexico). A volume/surface area ratio was settled at 9 mL/cm^2^. Each specimen was placed in a sealed polyethylene (PE) container with the proper immersion media. The containers were exposed to an accelerated physiological environment of 37 °C and 120 rpm during the corresponding immersion period. To provide these conditions, a general-purpose incubator (Sheldon Manufacturing Inc., Cornelius, OR, USA) was used. After the specified immersion period, the specimens were removed from the media and washed with distilled water. Then, the specimens were dried with compressed air and individually stored in PE containers with silica gel beads (desiccant) under a vacuum environment for 15 days. The desiccant was replaced every two days. Finally, the mass of the dried specimens was measured.

### 2.5. Crystallinity Measurements

Degradation of the scaffolds was followed through the application of FTIR, TGA, and DSC techniques. Infrared spectra of raw PLA and the printed scaffolds before and after the degradation test were collected using a Perkin Elmer FTIR (Model Frontier, Waltham, MA, USA) with a universal attenuated total reflectance (UATR) polarization accessory used to carry out the IR analysis, in absorbance mode with a resolution of 4 cm^−1^ and eight scans per sample in the mid-IR region of 4000–400 cm^−1^. The procedure consisted of placing the PLA samples on the ZnSe-diamond crystal of the UATR, compressed enough to guarantee a good contact among the sample and the incident IR beam. The influence of cooling on structure and degradation was investigated by comparing the spectral differences and using a normalized baseline at 956 cm^−1^. Spectra software was used for peak measurements and fitting.

TGA analyses were carried out in a Perkin Elmer TGA (Model 8000, Waltham, MA, USA). From each sample (approx. 5–10 mg) were placed in ceramic baskets and heated under a dry nitrogen gas flow rate of 40 mL/min, at 10 °C/min from 30 to 400 °C; temperature at which 95% of the mass was volatilized was recorded.

The glass transition and melting temperatures (*T*_g_–*T*_m_), heat of fusion (Δ*H*), and crystallinity ($X_{C}$) were obtained from the printed scaffolds before and after the degradation test using a Perkin Elmer DSC instrument (Model 8000, Waltham, MA, USA). All of the experiments were performed under a nitrogen atmosphere with a flow rate of 20 mL/min. The samples were prepared by sealing in an aluminum pan with between 5–10 mg of the printed scaffold, previously washed with distilled water and dried with pressurized air. In order to erase thermal history, the following procedure was followed: (a) heating up from 30 to 220 °C, (b) maintaining temperature at 220 °C for 3 min and (c) cooling down to 30 °C. For the second heating, the samples were held for 5 min at 30 °C, and then subsequently heated from 30 to 220 °C at 20 °C/min. Crystallinity was calculated using the following Equation (1):

(1) $$X_{C}=\frac{\Delta H_{S}}{\Delta H_{O}}$$

where $\Delta H_{S}$ is the heat of fusion for the sample and $\Delta H_{O}$ is the theoretical heat of fusion for 100% crystalline PLA, taken as 93 J/g [39].

## 3. Results

### 3.1. Manufacturing Process

**Figure 2:**
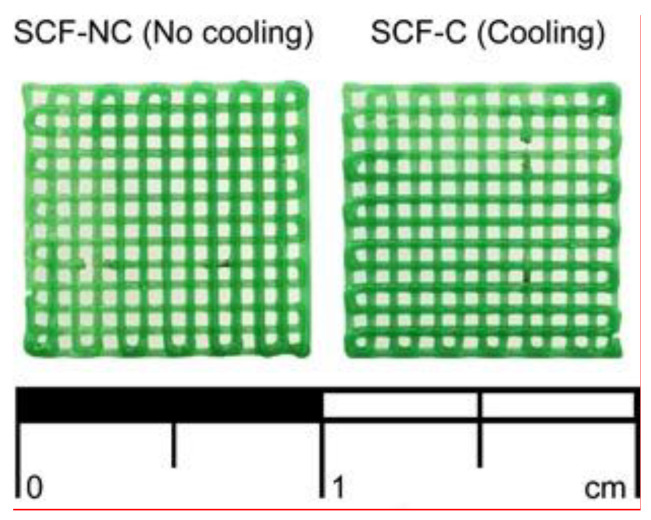
Scaffolds without printed and with the use of cooling stage (SCF-NC (no cooling) and SCF-C (cooling) specimens, respectively).

A total of 48 scaffolds were printed, 24 using the (SCF-C group) and 24 with no cooling (SCF-NC). The physical appearance was the same for both groups with a bright green coloration at the surface (see Figure 2).

### 3.2. Thermal Analysis of Manufacturing Process

**Figure 3:**
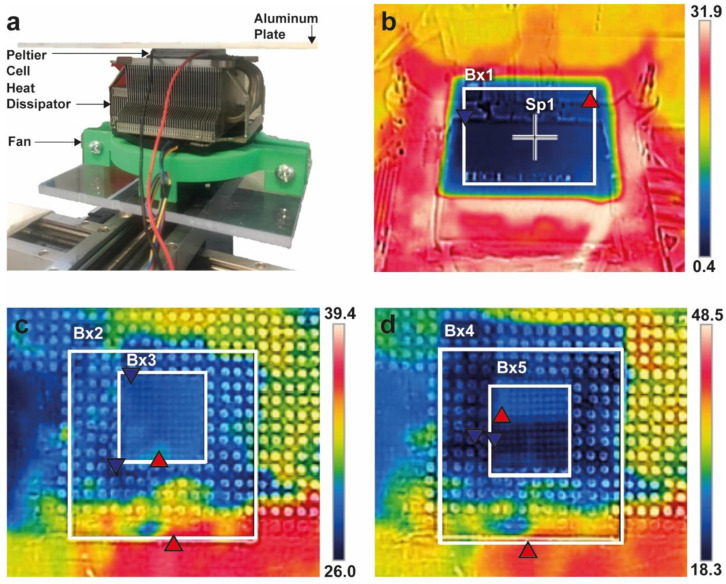
Temperature measurement analysis scheme (blue and red triangles indicate the lower and higher temperature zones inside the analyzed area). (**a**) Structure of the cooling system. It is composed of a Peltier cell controlled by Arduino, a heat sink, a fan, and an aluminum bed of 1/8 in., as the printing bed. (**b**) Peltier cell turned on. The local average temperature in zone Bx1 was 2.5 °C and the point Sp1 registered 2.3 °C. (**c**) SCF-NC printing. The temperature in the scaffold area (Bx3 zone), with the cooling system, turned off was 27.5 °C. (**d**) SCF-C printing. When the cooling system was turned on, the temperature in the scaffold area (Bx5) decreased by 20%.

Prior to the printing of the scaffolds, the temperature measurement was made with the thermal camera. Figure 3a shows the structure and construction of the cooling system, and Figure 3b shows a thermal image with the Peltier cell turned on. Blue and red triangles indicate the lower and higher temperature zones inside the analyzed area.

The lowest point of the analyzed zone Bx1 in Figure 3b registered a temperature of −0.2 °C and the local average temperature of 2.5 °C. In the central zone of the Peltier cell (point Sp1), the temperature was 2.3 °C. A second thermographic capture was made during the printing of the scaffold with the cooling system turned off, as can be seen in Figure 3c. Two analyzed zones were located: the Bx2, which corresponds to the area that receives direct cooling of the Peltier cell, and the area Bx3, which corresponds to the printed scaffold. In Bx2, the local average temperature was 28.1 °C, while in the area of the scaffold, the local average temperature was 27.5 °C.

When the cooling system was turned on, a change in the temperature was observed in the same analyzed zones, as shown in Figure 3d. The Bx4 and Bx5 zones decreased to a local average temperature of 23.9 and 21.9 °C, which indicates a decrease of 15% and 20%, respectively.

### 3.3. Mass Loss of Scaffolds after the Immersion Test

**Figure 4:**
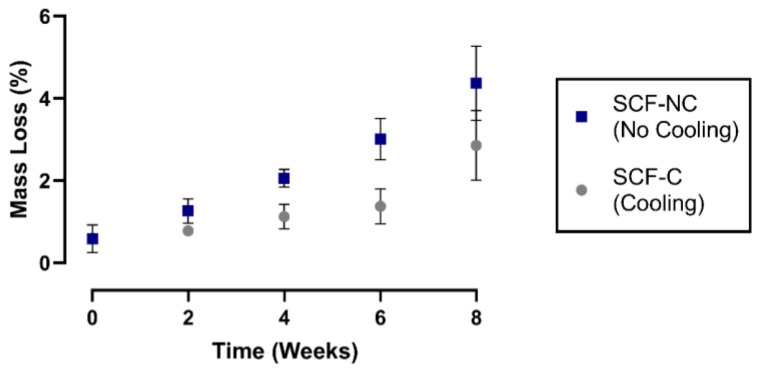
Mass loss percentage. At the end of the experiment, the SCF-NC samples lost 4.2% of their weight, while the SCF-C samples lost 2.5%.

**Figure 5:**
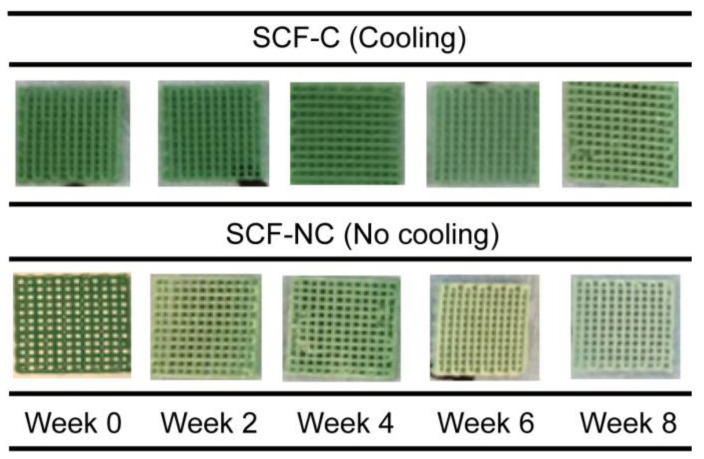
Photographs of samples after immersion. The color fading is a clue to the surface and erosion degradation induced by hydrolysis. The color fading is more evident in the SCF-NC specimens.

Figure 4 shows the mass loss percentage for the printed scaffolds through the immersion period. During week 2, only 1.3% and 0.7% were lost by the SCF-NC and SCF-C specimens, respectively. By week 8, the SCF-NC specimens reached the greatest loss of 4.2%, while the SCF-C specimens registered a loss of 2.5%. Moreover, it was observed that the SCF-NC group had faded from the bright green appearance by the end of the experiment. This effect was also observed to a lesser extent in the SCF-C specimens through the immersion time period, as shown in Figure 5.

### 3.4. Crystallinity Measurements

**Figure 6:**
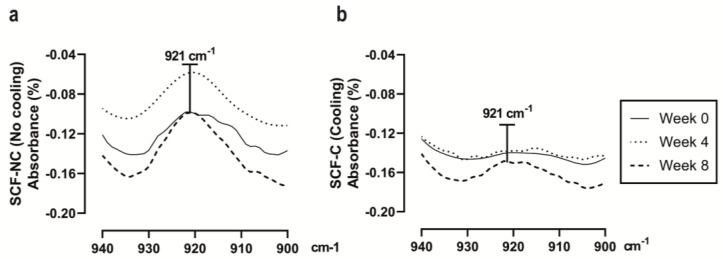
FTIR spectra. (**a**) the SCF-NC group from 930 to 910 cm^−1^. At the end of the immersion test, an increment of 76% in the peak area and height at 921 cm^−1^ was observed; (**b**) the SCF-C group from 930 to 910 cm^−1^. An increment of 139% was reached for week 8 at 921 cm^−1^. In both cases, the peaks increased with the immersion period.

**Figure 7:**
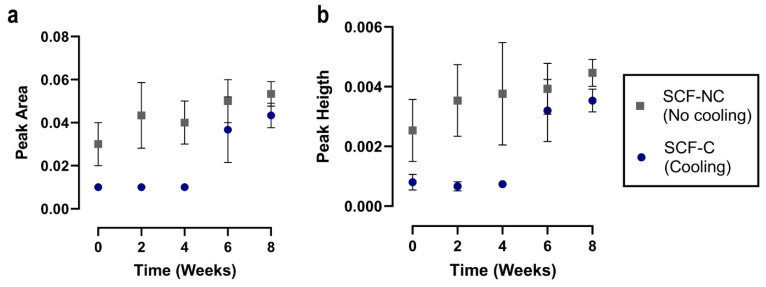
Peak area at 921 cm^−1^ during the immersion period. (**a**) A constant increment was observed for the SCF-NC group. For the SCF-C group, the increase was evident until week 6. (**b**) Similar behavior and increments were observed in the peak height. At the end of the experiment, the peaks showed increments of 76% and 136% for the SCF-NC and the SCF-C groups, respectively.

Figure 6 shows the FTIR spectra of the SCF-NC and the SCF-C scaffolds in the region of 930 to 910 cm^−1^. The absorption band at 921 cm^−1^ has been attributed in the literature to the flexural C–H bond vibration and is representative of the crystalline structure of PLA [6]. Because of this, measurements of peak area and peak height in the absorption band was monitored using the Perkin Elmer Spectrum v10 STD Software (Waltham, MA, USA). Figure 7 shows the measurements for peak at 921 cm^−1^. Prior to the immersion period (week 0), the SCF-NC group exhibited a peak 300% bigger in height than that of SCF-C in the absorption band at 921 cm^−1^. For the SCF-NC group, the increase in peak area and peak height were constant until week 4, when a slight decrease was observed. Then, at week 6, the peaks kept increasing until week 8, where an increment of 76% with respect to week 0 was reached. 

For the SCF-C group, there was a two-phase increase. From week 0 to 4, the area and height of the peaks were almost the same. After this, a significant increment was presented at week 6. By week 8, an increment of 139% was observed with respect to week 0. In both sample groups, the peaks increased with immersion time, but the SCF-NC group always showed larger peak areas and peak heights than the SCF-C group. The same phenomenon of the increase with immersion period is observed for both groups. By week 8, the intensity of the peak in the SCF-NC group is more notorious than SCF-C group. The increase in the height of the peak at 921 cm^−1^ suggests an increase in crystallinity of the samples, as was observed in other studies where the same band was used as reference to calculate the increase in crystallinity after thermal treatments [6].

**Figure 8:**
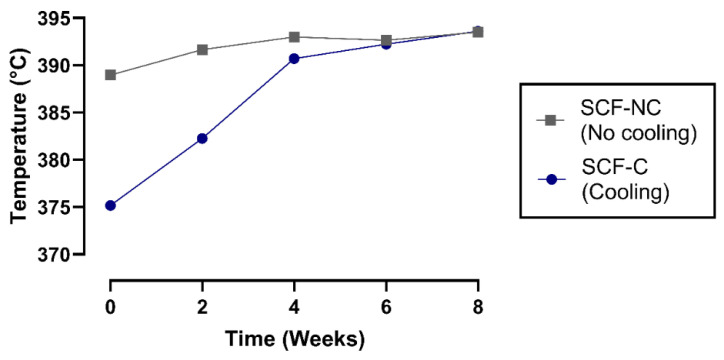
Decomposition temperature of scaffolds. An increase in the decomposition temperature [T_95_] with time was observed for the SCF-NC and SCF-C groups. The increase in decomposition temperature was 4.52 °C and 18.43 °C for the SCF-NC and SCF-C groups, respectively.

The decomposition temperature, which is related to the thermal stability of PLA, was obtained through TGA. That value, defined in the study as T_95_, is the temperature at which 95% of the total mass is volatilized [39]. Figure 8 shows the decomposition temperature of the SCF-NC and SCF-C specimens during the period of the immersion time. In the SCF-NC group, T_95_ increased by 4.52 °C from week 0 to week 8. The increment was slight during the immersion period. In the SCF-C specimens, the increment for T_95_ appeared to occur in two phases and was more evident from week 0 to 4. After this period, the increment was slight, and T_95_ reached 18.43 °C higher by week 8 with respect to week 0. The increment of T_95_ could be a clue of the increase in the crystalline phase of samples, which leads to more energy required to volatilize them.

**Figure 9:**
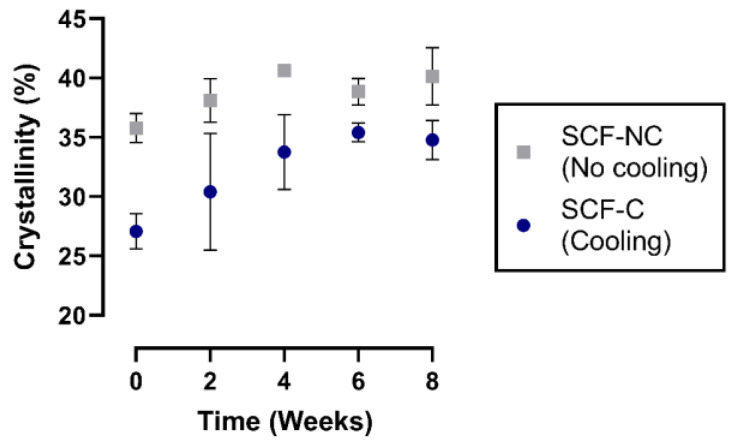
Crystallinity percentage of the scaffolds. In both instances, the degree of crystallinity increases, 14.3% for the SCF-C group, and 5.2% for the SCF-NC group by week 8.

The degree of crystallinity was obtained based on DSC for the SCF-NC and SCF-C groups at weeks 0, 2, 4, 6, and 8 (see Figure 9). For week 2 of the immersion period, the crystallinities calculated were 38.1% and 30.4% for the SCF-NC and SCF-C groups, respectively. By week 8, the groups presented an increment of crystallinity of 14.3% for the SCF-C group, and 5.2% for the SCF-NC group with respect to week 2.

## 4. Discussion

The work presented here evaluated the influence of temperature provided by a custom-made cooling system on PLA scaffolds. In order to analyze the effect of the cooling bed on the degradation properties of scaffolds, an immersion test was conducted to monitor changes in crystallinity. A higher increase in crystallinity and mass loss rate was found in the SCF-NC group, which indicates that the bed temperature is a significant manufacturing variable that could be used as a key for establishing resorption characteristics.

### 4.1. Thermal Analysis of Manufacturing Process

In the processing of PLA, there are studies reporting the influence of thermal fields on the mechanical properties and crystallinity [14,34,35,40]. In this study, the high level of crystallinity can be attributed to the absence of cooling in the SCF-NC group as extruded fiber is deposited on the bed (see Figure 3). During the printing, the fast cooling induced in the SCF-C group did not allow the polymer chains to rearrange into a crystalline structure. For that reason, samples of week 0 in SCF-NC group showed both, a higher crystallinity and higher peaks heights in bands at 921 cm^−1^ compared to the SCF-C samples (see Figure 6 and Figure 9). Other studies showed an increase not only in the band at 921 cm^−1^ but also in the bands at 1207 and 1760 cm^−1^ after thermal treatments, affecting thus the crystallinity of PLA [40,41] These bands are related to the C-O stretching components, nevertheless in this study, differences in the peaks were not detected.

### 4.2. Mass Loss of Scaffolds after Immersion Test

The study of the degradation kinetics of PLA was conducted through prolonged hydrolysis. This involves a chemical degradation process in which an aqueous medium diffuses into the polymeric material, while oligomeric products diffuse out. Diffusion inside the polymeric matrix produces scission of the chemical bonds and converts the very long chains into shorter, more water-soluble fragments [39]. Moreover, this phenomenon is appreciable through the surface and bulk erosion, which causes degradation on the surface and a loss of mass, respectively. In this study, the loss of mass was not accelerated as expected, but during immersion, the SCF-NC group revealed bulk erosion due to the fading of the bright green appearance obtained after the initial printing. This effect was observed as a minor effect in the SCF-C specimens, as shown in Figure 4.

The mass loss in the SCF-NC and SCF-C groups showed an increasing tendency with immersion time. Moreover, it was observed that the SCF-NC specimens degraded faster than the SCF-C group. This phenomenon could be explained because of the initial degree of crystallinity, which was higher for the SCF-NC group after the printing (see Figure 9). According to other studies, the initial degree of crystallinity plays an important role in the alkaline hydrolyzibility of PLA, therefore affecting the degradation rate [42].

Gorrasi et al. analyzed the influence of D-lactide content on the hydrolysis of PLA films. They reported that amorphous samples (12% of D-lactide content) showed a faster rate in weight loss than a crystallized (2% of D-lactide content) sample during immersion in distilled water [6]. On the other hand, Vasanthan et al. demonstrated two important facts in an accelerated degradation test for poly(L-lactic acid) (PLLA) films with 6% of D-lactide content and different crystallinity: (a) the maximum weight loss increases under alkaline hydrolysis conditions with more initial crystallinity and (b) the material between spherulites was etched away by the loss of spherulitic boundaries [39]. The contradictory trends shown [6,39] could be attributed to different testing conditions (such as media and temperature) and/or different D-lactide content of samples. Our findings revealed similar results to those reported by Vasanthan et al. in regards to the influence of initial crystallinity and weight loss. The SCF-NC group with around 36% of initial crystallinity showed the higher increase in weight loss with respect to the SCF-C group (27% initial crystallinity).

### 4.3. Crystallinity Measurements after Immersion

FTIR analysis showed that the peak area and peak height in the absorption band at 921 cm^−1^ were larger for the SCF-NC group than the SCF-C group prior to the immersion period. This difference in peaks is explained because of the effect produced by using the cooling system. During the printing, fast cooling generated by the Peltier cell in the SCF-C group did not allow the polymer chains to rearrange into a crystalline structure. Because of that, a difference in crystallinity between the two groups was expected after the printing. Moreover, the absence of cooling will produce a higher crystallinity in the SCF-NC group. Those assumptions were corroborated when comparing the bands between groups. In this way, measurements of the absorption band at 921 cm^−1^ served as an initial marker to validate the effect of cooling in the printed scaffolds and to monitor the increase in crystallinity during the immersion period.

TGA also revealed an incremental tendency for the temperature required to volatilize 95% of the mass of the scaffold in the SCF-NC and SCF-C groups during the period of immersion. This trend was produced due the continuing increase in the crystallinity for each group over time, indicated by an increase in the energy required to thermally degrade the samples. 

Finally, DSC corroborated the statements about the different levels of crystallinity observed with the FTIR and TGA measurements. The increment in crystallinity could be explained due to two factors occurring in the amorphous zones of the PLA scaffolds. First, the erosion of some of the amorphous parts that produces a loss of molecular fragments. This leads to an increase in the crystalline/amorphous ratio. Second, the crystallization of degraded amorphous phases which occurs, resulting in the presence of new crystals. That phenomenon of crystallization takes place due to the constant temperature in the incubator (37 °C), from which, the local mobility of PLA chains can be enhanced by low molecular weight fragments and the plasticity effect of water as was observed previously in similar studies [6,7]. The presence of new crystals not only changes the crystalline/amorphous ratio, but also could have an effect on the degradation because they will be more difficult to erode [7].

The same effect of increasing crystallinity was observed in other studies where PCL scaffolds were immersed in an alkaline medium for six weeks. The increase was attributed to the combined effect of removal of the amorphous regions and the annealing effect of the incubator temperature at 37 °C [6]. In addition, similar studies conducted with different PLA grades and crystallinities have also followed this pattern of increasing crystallinity [7].

### 4.4. Relationship between 3D Printing Process Parameters and PLA Scaffold Degradation

**Figure 10:**
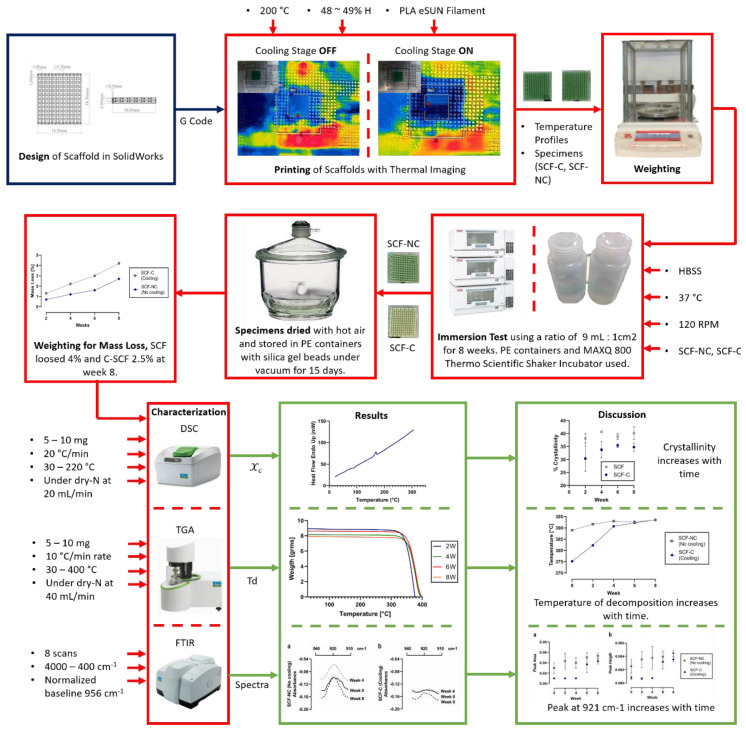
Schematic description of the manufacturing and characterization processes of the Polylactide acid (PLA) scaffolds.

In the context of the scaffold printing processes, significant manufacturing variables, such as bed temperature and crystallinity, were established. These specifications were correlated to the basic properties of the 3D printed scaffolds, which were derived from the complete array of process parameters. Figure 10 shows a schematic relationship between the process parameters, the basic properties, and the scaffold specifications. This work reports the consequential effects of controlling and monitoring the bed temperature and the crystallinity of the printed scaffold, similar to other studies previously presented in Table 1. As the printing and cooling system processes are further refined, a study with additional variations in the bed temperature and longer immersion periods will be required.

## 5. Conclusions and Future Work

Tissue engineering is experiencing a period of enhanced innovation. The implementation of new manufacturing techniques, for example, has allowed the development of various initiatives to treat a wide variety of medical applications, including critical size defects in long bones. Following this path, the cooling system developed in this study for a custom-made FDM printer shows promising potential in terms of better control of mechanical and chemical properties associated to PLA scaffolds.

After the implementation of the described cooling system, the degradation rate of two groups of PLA scaffolds manufactured at different printing bed temperatures was evaluated. Immersion tests for both groups were performed during eight weeks in HBSS solution in a controlled temperature environment and agitation speed (37 °C–120 rpm). The results showed that the SCF-NC group (printing bed at 28.1 °C) presented a higher mass loss at the end of the immersion period than the SCF-C group (printing bed at 21.9 °C). This phenomenon was attributable due two phenomena. First, because of to the mechanism associated with alkaline hydrolysis, where the higher level of crystallinity obtained in the SCF-NC group after printing induced higher rates of mass loss, in agreement with similar studies [39]. Second, because during immersion an erosion of some of amorphous parts could produce a loss of molecular fragments. This leads to an increase in the crystalline/amorphous ratio.

Fast cooling after the printing of the SCF-C specimens did not allow the polymer chains to rearrange into a crystalline structure. For this reason, FTIR was used to monitor the absorption bands at 921 cm^−1^, which is representative of the degree of crystalline structure in PLA [6,39,40,41]. This band was observed to be slightly smaller in the SCF-C group than in the SCF-NC group after printing the scaffold. In addition, the bands increased with immersion time, which suggests that the degree of crystallinity also increased. Increments in the absorption band at 921 cm^−1^ were calculated to be 76% and 139% for the SCF-NC and SCF-C groups, respectively. This was corroborated by the increments in crystallinity measured by DSC (5.2% and 14.3% for the SCF-NC and SCF-C groups, respectively), and was in accordance with the increments in the temperature for thermal decomposition (T_95_) measured by the TGA assay (4.54 °C for SCF-NC and 11.35 °C for SCF-C).

The increase in crystallinity that developed over time was explained due to the erosion of some amorphous parts in the printed scaffold that produces a loss of small molecular fragments. This leads to an increase in the crystalline/amorphous ratio, as was previously explained. At the same time, the crystallization of degraded amorphous phases could initiate the presence of new crystals due to the local mobility of PLA chains and the plasticizing effect of the water, according to other studies [6,7,39,43].

PLA filament with the same chemical composition as the one reported here has been studied to analyze its cytotoxicity effect in previously published studies, showing good cell viability [14,15,16]. Those results provide information about the potential use of the material for medical use, mainly for bone tissue. However, further experimentation will be required to compare the performance and crystallinity behavior of the commercial PLA with medical grade PLA during a period of immersion.

Concerning future work, optical micrographs of spherulites of the specimens from both groups, and the manufacture of scaffolds using a wide variation of temperature in the printing beds will be needed. More changes in the printing bed temperature could provide adequate information to know if the use of a cooling system produces a direct effect on the degradation of the printed scaffolds.

## Figures and Tables

**Table 1:** Comparative table of the related work in regards characterization on 3D printed scaffolds.

Material	Technique	Characterization Parameters	Ref
Poly(L-lactide) (PLLA) poly(L-lactide-co-ε-caprolactone) (PCLA) poly(L-lactide-co-glycolide) (PLGA) poly(D,L-lactide-co-glycolide) (PDLGA)	Direct extrusion-based 3D printing	Crystallinity (%), before and after printing. Degradation profile within 8 h after printing	[28]
Polycaprolactone (PCL)	Extrusion based cryogenic 3D printing (ECP) (−20 °C) and subsequent freeze-drying approaches	Crystallinity (%) after the printing Degradation profile within 12 h after printing	[29]
Na2O–CaO–MgO–P_2_O_5_ Bioglass reinforced β-TCP	Inkjet 3D printing technology	Degradation profile after soaking the scaffolds within 35 days after printing Degradation profile considering each time point within 7 days.	[30]
Polycaprolactone (PCL)	3D-Bioplotter	Degradation profile considering each time point within 7 days.	[31]
Polylactic acid (PLA)/ Polyethylene glycol (PEG)/ (nano hydroxyapatite (nHA)/Dexamethasone (Dex)	Fused deposition modeling (FDM) process	Crystallinity (%) before and after the degradation experiment within 8 weeks Degradation process after heating process within 8 weeks after printing	[32]
Poly-Ether-Ether-Ketone (PEEK)	Temperature control system into fused deposition modeling (FDM) process	Crystallinity (%) results of different PEEK samples with different heat treatment methods	[33]
Polylactic acid (PLA)	Continuous heat transfer during fused deposition modeling (FDM) process	Crystallinity (%) for in-process heat treatment samples	[34]
Polylactic acid (PLA)	Fused deposition modeling (FDM) process	Degradation profile before and after printing Crystallinity (%) before and after printing	[14]
Polycaprolactone (PCL)	BioExtruder Extrusion-based additive manufacturing (AM)	Enzymatic degradation profile after printing within 30 days Crystallinity (%) during degradation period	[35]
Polylactic acid (PLA)	Fused deposition modeling (FDM) process	Crystallinity (%) during different thermal printing conditions (different print temperature, bed temperature, and annealing temperature) and injection-molded samples	[36]

**Table 2:** Data sheet of the Polylactide acid (PLA) filament employed [38].

Property	Value
Density (kg/m^3^)	1.20–1.25
Melting Point (°C)	190–220
Tensile Yield Strength (MPa)	65.63
Flexural Strength (MPa)	65.02
Flexural Modulus (MPa)	2504.4
Glass Transition Temperature (°C)	56–60 *
Crystallization Temperature (°C)	130–173 **

* Based on ASTM E1356; ** Based on ASTM D3418.

## Nomenclature

Symbol	Description
PLA	Polylactic acid
PLLA	Poly (L-lactide acid)
FDM	Fused Deposition Modeling
HBSS	Hank´s Base Solution
AM	Additive Manufacturing
CAD	Computer Aided Design
PEEK	Poly-Ether-Ether-Ketone
PID	Proportional-Integrative-Derivative control
SCF-NC	Scaffolds printed with No-Cooling
SCF-C	Scaffolds printed with Cooling
PE	Polyethylene
TGA	Thermo Gravimetric Analysis
FTIR	Fourier Transformed Infrared spectroscopy
DSC	Differential Scanning Calorimetry
$X_{C}$	Crystallinity
T_g_	Glass transition temperature
T_m_	Melting temperature
Δ*H*	Heat of fusion
Δ*H_o_*	Theoretical heat of fusion for 100% crystalline PLA
Δ*H_S_*	Heat of fusion for sample
